# In Vivo Differentiation of Cutaneous Melanoma From Benign Nevi With Dual‐Modal System of Optical Coherence Tomography and Raman Spectroscopy

**DOI:** 10.1002/jbio.70040

**Published:** 2025-04-21

**Authors:** Di Wu, Anatoly Fedorov Kukk, Rüdiger Panzer, Steffen Emmert, Bernhard Roth

**Affiliations:** ^1^ Hannover Centre for Optical Technologies Leibniz University Hannover Hanover Germany; ^2^ University Medical Center Rostock Rostock Germany; ^3^ Cluster of Excellence PhoenixD Leibniz University Hannover Hannover Germany

**Keywords:** attenuation coefficient, melanoma diagnosis, multimodal noninvasive cancer diagnosis, optical coherence tomography, Raman spectroscopy

## Abstract

A multimodal method comprising optical imaging using OCT and molecular detection using Raman spectroscopy was developed to explore its capability for noninvasive differentiation between melanoma skin cancer and benign skin lesions. Key OCT parameters like the attenuation coefficient, *R* ^2^, and RMSE, extracted through exponential fitting, were incorporated into machine learning, achieving 96.9% accuracy and an AUC‐ROC of 0.99 in 10‐fold cross‐validation. Raman spectroscopy revealed differences in carotenoid, amide‐I, and CH_2_–CH_3_ structures between melanoma and nevi, supporting the OCT findings. Autofluorescence background intensity variations further distinguished lesion types and enhanced lesion assessment. Future work will include the investigation of larger patient groups and the combination of both data sets in a combined algorithm. Also, the integration of both modalities and the developed method with photoacoustic tomography and high‐frequency ultrasound appears beneficial toward achieving an optical biopsy of melanoma skin cancer and improving diagnostics.

## Graphical abstract

An innovative method for noninvasive melanoma diagnosis using the attenuation coefficient from OCT scans and machine learning has been proposed, supporting small sample sizes. When combined with Raman spectroscopy, it can further enhance accuracy.

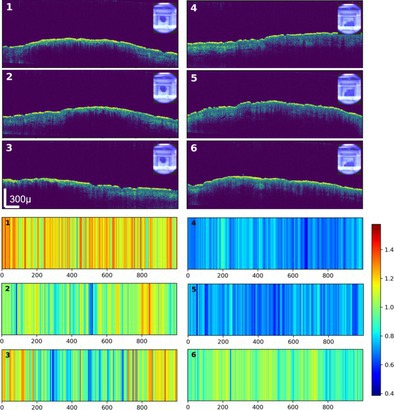

## 1 Introduction

Skin cancer is the most common type of cancer worldwide. Although melanoma accounts for only around 10% of all skin cancers, more than half of skin cancer‐related deaths are attributed to melanoma, and the number of new cases has been steadily increasing. It is estimated that the number of newly diagnosed melanoma cases increased by 7.3%, and the number of resulting deaths by 3.8% in 2024 [1]. Patients with melanoma detected at an early stage are expected to have a five‐year survival rate of more than 99%; this rate drops to 74% when the disease reaches lymph nodes and 35% when it metastasizes to distant organs [1]. Even though these numbers differ between different studies, they all emphasize the dramatic change in survival prognosis between early and late stage diagnosis cases. Unfortunately, due to the morphological similarities between melanomas and benign entities such as nevi, congenital birthmarks, or acquired lesions, it is easy to overlook a melanoma and mistake it for a birthmark or mole, thereby delaying the most appropriate treatment and increasing the risk of death.

The initial diagnosis of cutaneous melanoma is usually made by visual inspection or complementary dermoscopy in a clinic. If a skin spot is suspected to be a melanoma, the suspicious area will be excised and sent for histopathological evaluation. However, the accuracy of these visual diagnoses is highly dependent on the experience and expertise of the diagnosing physician. For the case of more inexperienced physicians, patients are possibly misdiagnosed with false positives, leading to unnecessary excision surgery, or false negatives, delaying treatment. Consequently, and especially given the rapidly increasing incidence of melanoma, impartial, credible, accurate, and noninvasive companion diagnostic techniques are needed to improve clinical decision‐making and increase the willingness of patients to undergo screening.

By measuring the echo time delay and intensity of backscattered light, optical coherence tomography (OCT) generates noninvasive high‐resolution real‐time images that resemble histological sections. In recent years, OCT has been extensively studied for its potential in diagnosing various skin cancers [2, 3, 4]. Gambichler et al. used OCT to characterize benign and malignant melanocytic skin lesions in vivo and found that malignant melanoma (MM) often exhibited significant architectural disorganization and rarely displayed a clear dermal–epidermal junction compared with benign nevi (BN) [5]. Jørgensen et al. studied the OCT images of 41 basal cell carcinomas (BCC) and 37 actinic keratosis (AK) lesions from 34 patients. When using multiple image features for machine learning classification, the accuracy of 73% (AK) and 81% (BCC) was achieved, respectively [6]. Although these numbers can still be improved, the potential of using machine learning is demonstrated.

In addition to OCT, Raman spectroscopy has shown promising potential for the diagnosis of skin cancer [7, 8]. Patil et al. combined OCT and Raman spectroscopy to simultaneously characterize the morphology and biochemistry of skin cancer [9]. Varkentin et al. and Kukk et al. introduced trimodal systems that integrate photoacoustic imaging [10] or ultrasound imaging [11] on the OCT‐Raman system for in vivo skin cancer screening, which enable deeper morphological images when the lesion depth exceeds the OCT imaging depth. You et al. combined OCT and Raman spectroscopy, extracted cell volume, density, surface roughness, average intensity, and internal intensity standard deviation from 3D OCT images, and removed the fluorescent component from Raman spectra for machine learning. An accuracy of 85% was achieved in classifying five types of skin cells [12]. However, whether OCT is used alone or in combination with other modalities to diagnose skin cancer, existing research work has only used OCT for morphological characterization, judging by features such as surface roughness, penetration depth, or specific structures of different lesions.

In this study, we explored a novel approach of utilizing OCT with assisted Raman spectroscopy for the noninvasive in vivo melanoma diagnosis. According to the Lambert–Beer law, the attenuation of each A‐scan signal in the OCT B‐scan was fitted to obtain the attenuation coefficients of different scan areas. These attenuation coefficients encompass the absorption and scattering characteristics of the tissue; variations of attenuation coefficients in different areas reflect differences in the tissue structures of various lesions. The attenuation coefficients, along with the coefficient of determination (*R* ^2^) and root mean square error (RMSE) were used as variables of machine learning algorithms to distinguish between suspected melanoma and healthy lesions. The approach was tested on the patient with both melanoma and BN, with a 96.9% accuracy in correctly identifying the lesion types across 1200 A‐scans. When the results of all A‐scans within each lesion were combined, MM and BN were both accurately predicted. Additionally, Raman spectroscopy was integrated into the OCT device to achieve multimodal optical analysis and obtain the biochemical characteristics of different skin lesions, which helps to comprehensively evaluate key diagnostic parameters for distinguishing MM from BN, thereby enhancing the overall diagnostic framework. In the next phase, we will aim to collect more cases of melanoma and other types of skin cancer to further develop and validate the proposed method. Our goal is to integrate both modalities into a unified machine learning algorithm, ultimately enabling noninvasive and more objective in vivo diagnosis of skin cancer, increasing patient acceptance and willingness of screening, and improving the accuracy of clinical diagnostics [13, 14].

## 2 Materials and Methods

### 2.1 Experimental Setup

**FIGURE 1:**
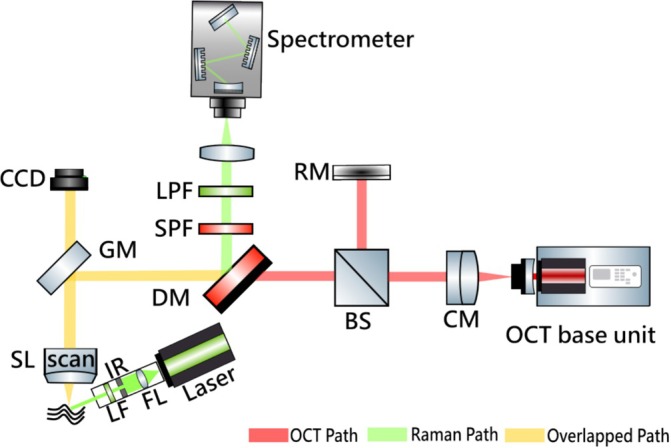
Sketch of the experimental setup. BS, beam splitter; CCD, charge‐coupled device; CM, collimator; DM, dichroic mirror; FL, focal lens; GM, galvo mirror; IR, iris; LF, laser filter; LPF, long pass filter; RM, reflector; SL, scan lens; SPF, short pass filter.

Figure 1 presents a schematic diagram of the system used in this work. The OCT part comprises a base unit (GAN621, Thorlabs, USA) and a scanning system (OCTP‐900, Thorlabs, USA); its wavelength is centered at around 930 nm. Real‐time OCT scanning is achieved by adjusting the angle of the galvo‐mirror. The scanning system was modified by incorporating a dichroic mirror (DM, edge wavelength 805 nm, DMLP805R, Thorlabs, USA) between the galvo‐mirror and the beam splitter, facilitating the connection to the Raman collection arm. This modification ensures the separation of OCT and Raman signals after passing through the DM, directing them to distinct collection devices and thereby enabling sequential OCT‐Raman measurements in vivo.

For Raman signal excitation, a 532 nm pulsed frequency‐doubled Nd:YAG laser (20 Hz repetition rate, 7 ns pulse duration, Ultra50, Quantel Laser, France) was used. The laser is transmitted through an optical fiber with a core diameter of 910 μm (MPH910L02, Thorlabs, USA) to a custom‐made collimation head [15]. A band‐pass filter (center wavelength 532 nm, bandwidth 4 nm, FLH05532‐4, Thorlabs, USA) is integrated at the end of the head to eliminate fluorescence and Raman signals generated before the laser reaches the sample.

Prior to focusing by a lens (focal length 60 mm, AC254‐060‐A‐ML, Thorlabs, USA), the scattered Raman signal passes through a short‐pass filter (edge wavelength 700 nm, FESH0700, Thorlabs, USA) and a long‐pass filter (edge wavelength 532 nm, LP03‐532RU, Semrock Inc., USA) to thoroughly filter out the OCT signal and Rayleigh scattered signal. The signal is then transferred to a spectrometer (Kymera 193i, Andor Technology Ltd., UK) equipped with a CCD camera (iDus‐LDC‐DD, Andor Technology Ltd., UK) and a grating (1200 L/mm, 600 nm blaze, MKS Instruments Inc., USA).

### 2.2 Patient Information and In Vivo Measurement

In this study, two skin lesions located in similar locations on the left and right thighs of a volunteer patient (male, 66 years old, Fitzpatrick Skin Type II) were evaluated in vivo to demonstrate the principle of our approach. To validate the experimental results, both lesions were surgically excised and subjected to histopathological examination to obtain a definitive clinical diagnosis. The lesion on the left thigh was confirmed to be a benign nevus, while the lesion on the right thigh was diagnosed as malignant in situ melanoma. The skin on the left and right thighs of the same patient is assumed to have the identical physiological structure, so all the differences between these two lesions are theoretically caused solely by the different biochemical properties of melanoma and nevus, eliminating potential variations due to different skin sites or inter‐patient variability. The performed measurements were approved by the Ethics Committee of the University Medical Center Rostock (A 2016–0115) and met the principles of the Declaration of Helsinki. Informed consent was obtained from the participant in this study, in oral and written form.

In the clinical measurements, each lesion underwent three OCT B‐scans at an A‐scan rate of 100 kHz. Each B‐scan captured 2634 pixels over a scale of 7–8 mm in the horizontal direction, with each pixel representing the average of three A‐scans. The duration of each B‐scan was below 1 s. Due to the use of low‐coherence light in OCT, the energy density remained consistently below the MPE level.

Then, three points were randomly selected from each lesion for Raman spectroscopy measurement to investigate the biochemical structural differences between BN and MM. The target skin area of 0.5 cm^2^ was irradiated with an average laser energy of 4.8 mJ in total. The exposure time for the lesion measurement was set to 10 s. Throughout the procedure, the irradiation density to which the patient was exposed was calculated to be below the maximum permissible exposure (MPE) value, ensuring safety during skin exposure [16, 17].

### 2.3 Attenuation Coefficient Fitting and Multivariate Statistical Analysis

The Lambert–Beer law describing light absorption in a medium can be expressed as follows:

(1) $$I\left(z\right)=I_{0}e^{-\mathrm{\mu z}}$$

Here, $I\left(z\right)$ is the signal intensity corresponding to the depth z. In OCT, since light undergoes two attenuation paths (one from the light source to the tissue depth and the other from the depth back to the detector), the equation can be rewritten as:

(2) $$I\left(z\right)=I_{0}e^{-2\mathrm{\mu z}}$$

In fact, Schmitt et al. also found that the backscattered power of low‐coherence reflection decays exponentially with depth at a rate of 2μ, where μ is the total attenuation coefficient accounting for absorption and scattering losses [18].

**FIGURE 2:**
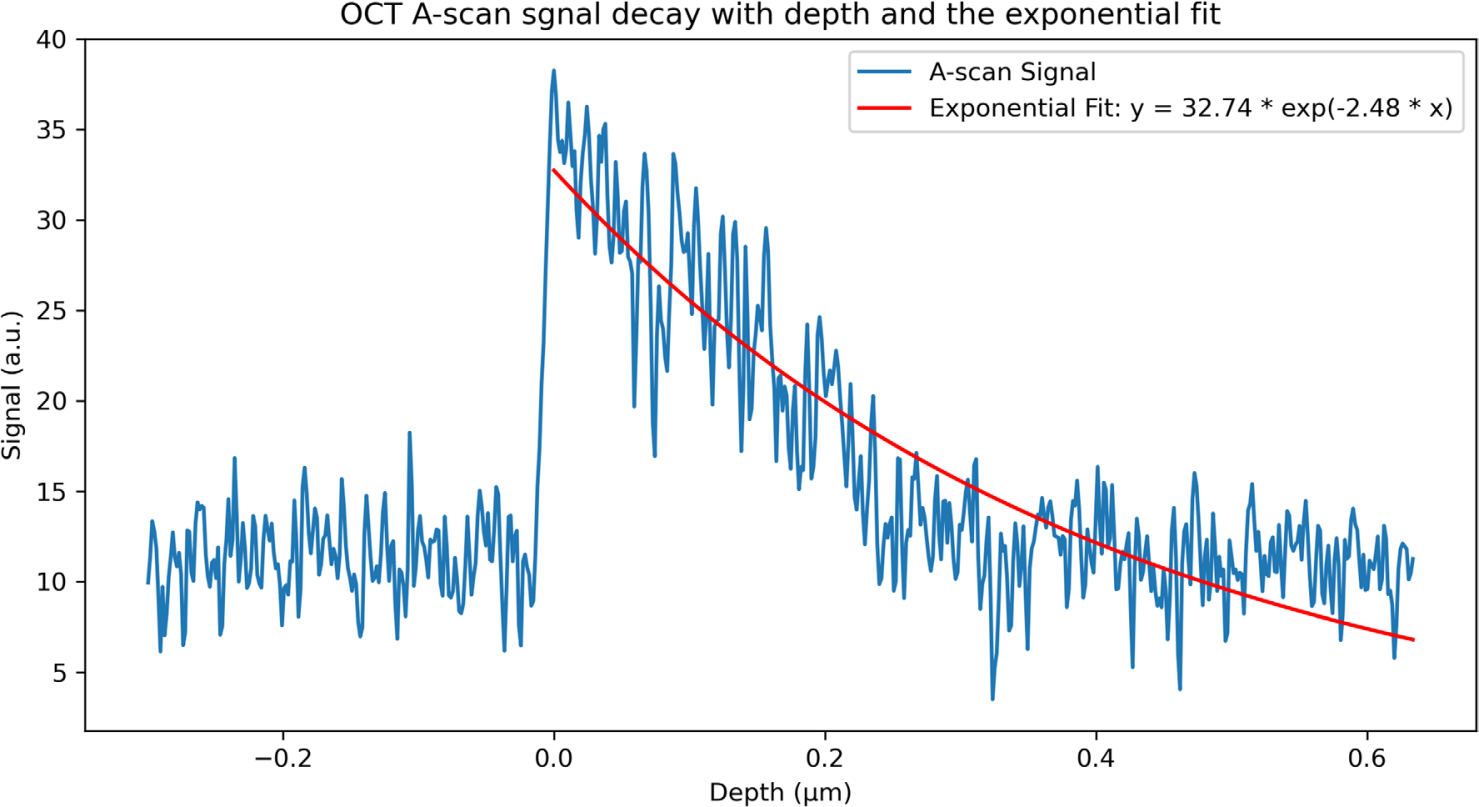
Typical OCT A‐scan signal, which has the maximum signal intensity on the sample surface. From here, an exponential decay fit is performed, and the fitting function obtained is shown in the legend. Based on the function, the total attenuation coefficient of this A‐scan can be calculated to be 1.24.

For each OCT A‐scan in our measurement, an exponential function $a\times e^{-2b}$ is defined to fit the decay with depth. The obtained parameter *b* is the attenuation coefficient. Then, the coefficient of determination and the RMSE of the fitted function are calculated. Figure 2 shows a typical OCT A‐scan signal and the fitted exponential function.

In this way, for each A‐scan, the corresponding attenuation coefficient, *R* ^2^, RMSE, and maximum signal intensity were obtained. To mitigate the impact of minor observational errors, the results from every five A‐scans were binned, and the averages and standard deviations of the aforementioned parameters of each bin were calculated and used as inputs for machine learning classification. Classification was performed using a support vector machine (SVM) with 10‐fold cross‐validation; the kernel function was set to linear, employing a linear kernel function and a regularization parameter of 1. For each cross‐validation, the area under the receiver operating characteristic curve (AUC‐ROC) score was computed; the ROC curves of all steps were interpolated, and the mean AUC along with its standard deviation across all folds were calculated.

As for the Raman data, all acquired Raman spectra were processed through smoothing and cosmic ray removal, followed by normalization within the fingerprint region of 800–1800 cm^−1^. Average spectra were plotted for BN and MMs. Twenty points were uniformly selected on these mean spectra to illustrate confidence intervals. Subsequently, a baseline correction method using asymmetrically reweighted penalized least squares smoothing was used to eliminate the autofluorescence background [19] and the difference between the mean spectra of the nevi and melanomas and the confidence intervals were calculated in order to identify the difference in the intensity of the corresponding Raman bands of malignant and benign lesions.

## 3 Results and Discussion

### 3.1 OCT Tomography Images

**FIGURE 3:**
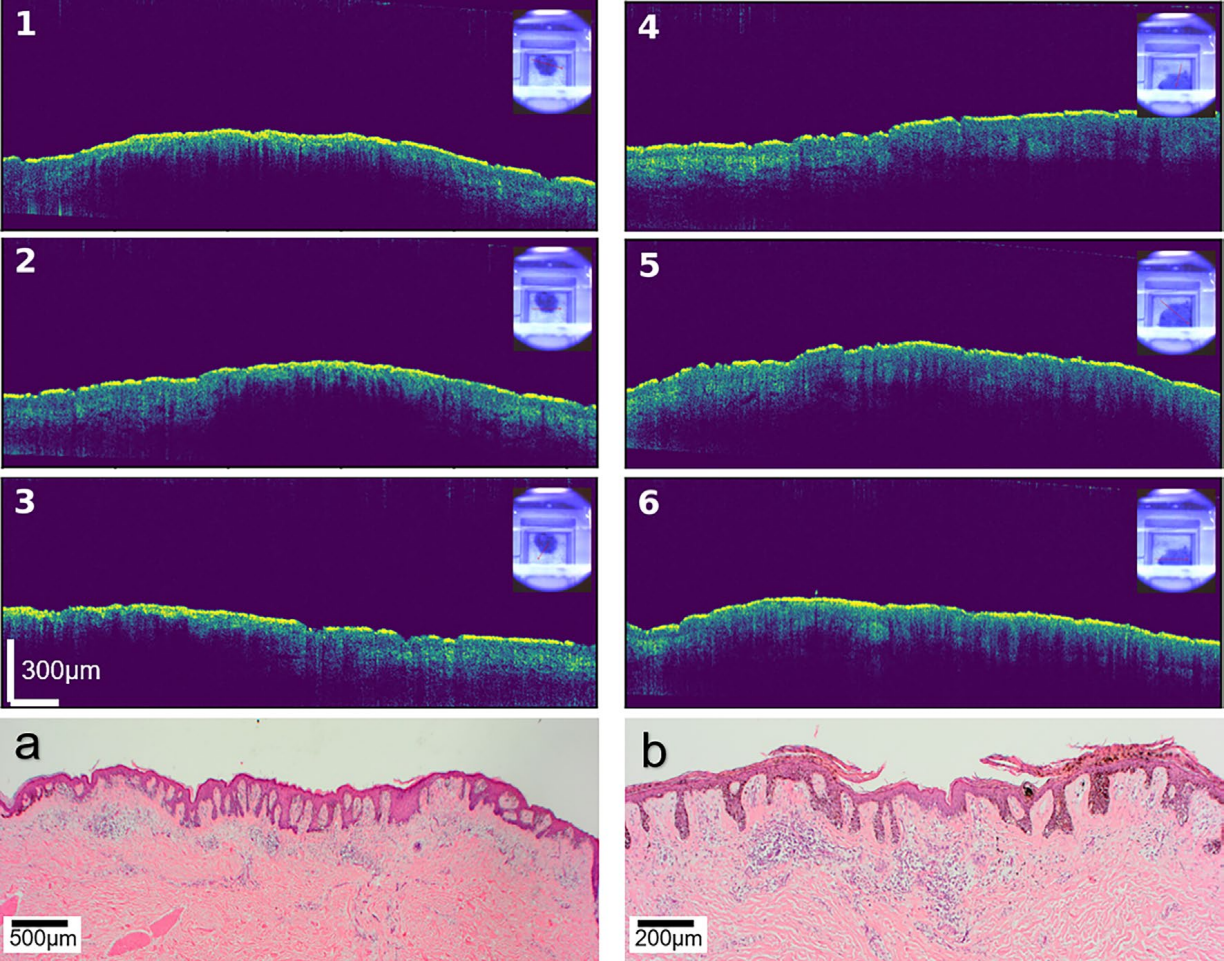
(a–f) OCT B‐scans of benign nevi and malignant melanomas as well as camera photographs, where (a–c) on the left side are benign nevi and (d–f) on the right side are melanoma. The lines with red arrow in camera images indicate the direction of the scan. All the OCT images have the same scalebar, to avoid repetition, it is shown only in (c). (g, h) show histopathological images of the measured nevi and melanoma, respectively.

The B‐scan results of OCT reveal the morphological characteristics of nevi and melanoma from the surface of the skin to a depth of approximately 0.3 mm subcutaneously. A very intuitive observation is that both the nevi and melanoma have a distinct difference in penetration depth from normal skin. When scanning the lesion area, the A‐scan signal is denser and shallower, whereas in the normal skin area, the signal is sparser and penetrates deeper, as shown in Figure 3.

In addition, it was found that the B‐scan images did not have a uniform penetration depth within the adjacent area, but rather showed a jagged or comb‐like depth of penetration, which is consistent with the “J‐shape” structure of the dermal‐epidermal junction shown in the histopathological images. The subcutaneous depths of them are also consistent in both OCT B‐scan and histopathological images, suggesting that they reflect the same structure.

Since the focus was only on the difference between BN and MM, for each B‐scan, only 1000 A‐scans of the lesion areas were selected. Afterward, for each A‐scan, the corresponding attenuation coefficient was obtained according to the fitting equation in Section 2.3.

**FIGURE 4:**
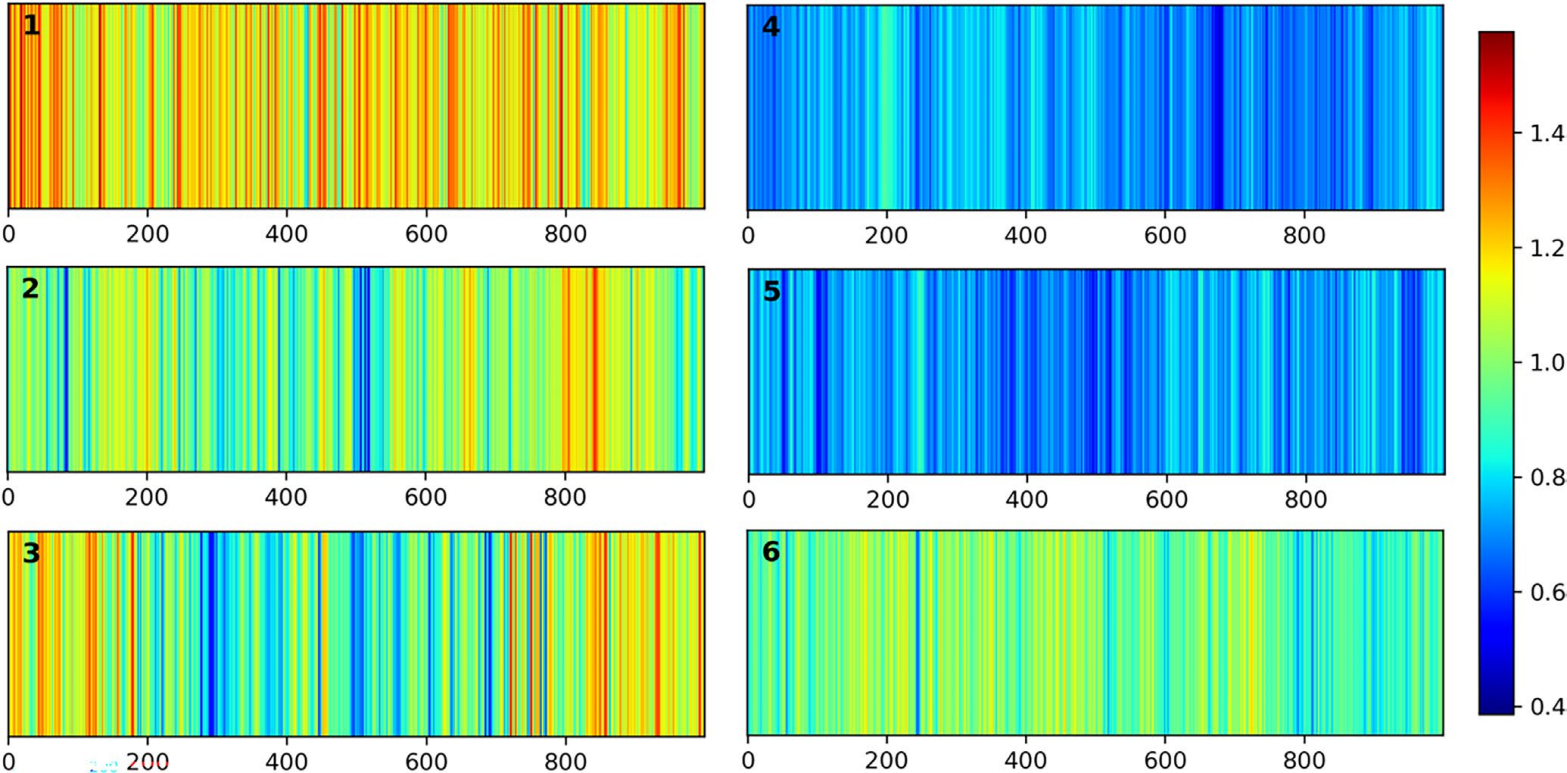
Heatmap of attenuation coefficients of 1000 A‐scan signals obtained after exponential decay fitting for the lesion area in the B‐scan, where (a–c) on the left side are benign nevi and (d–f) on the right side are melanomas, corresponding to Figure 3a–f, respectively. The *x* axis is the number of A‐scans, and the color coding on the right indicates the value of the attenuation coefficient in the figure.

In the scattering of biological tissues, the refractive index must be considered. Therefore, the attenuation coefficient obtained theoretically through fitting is effectively the product of the attenuation coefficient and the refractive index. The refractive index of biological tissues typically ranges from 1.33 to 1.55, which explains why the maximum value of the fitted attenuation coefficient in Figure 4 is approximately 1.6, rather than 1. The attenuation coefficients of the BN in Figure 4a–c are generally higher than those of the MMs in Figure 4d–f, indicating that the attenuation coefficient has the potential to serve as an important indicator for assessing the malignancy of a lesion.

### 3.2 Machine Learning Classification

The exponential decay fitting results of 1000 A‐scans of the lesion site for 6 measurements were binned in groups of 5 A‐scans, and the corresponding mean and standard deviation were taken as input parameters. Two hundred instances were obtained for each measured sample, and the sample size of the dataset was 1200. A total of 8 parameters were considered, namely the mean attenuation coefficient, mean *R* ^2^, mean RMSE, and mean maximum A‐scan signal intensity, as well as the standard deviation of their mean values in each bin.

**FIGURE 5:**
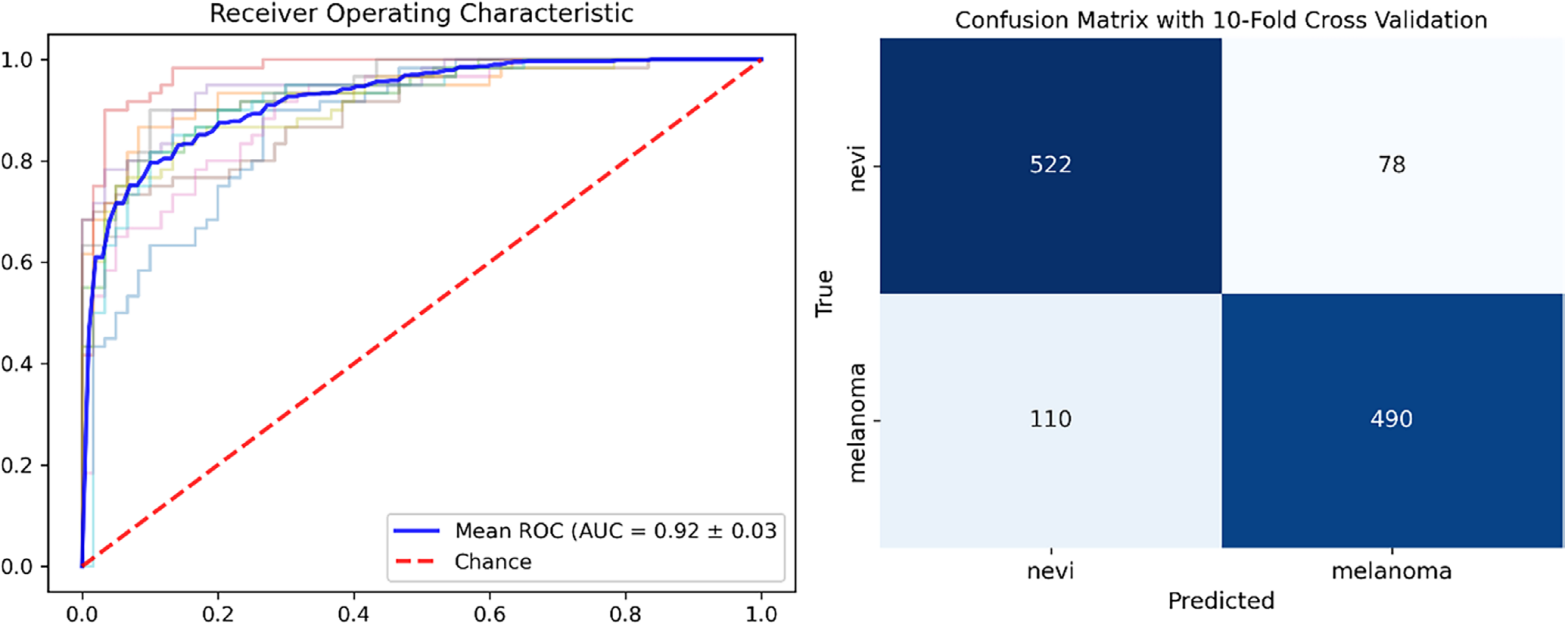
SVM classification statistics with 10‐fold cross‐validation, with only the mean attenuation coefficient and its standard deviation as input, yielded an average AUC‐ROC of 0.92 and a classification accuracy of 84.3%.

**FIGURE 6:**
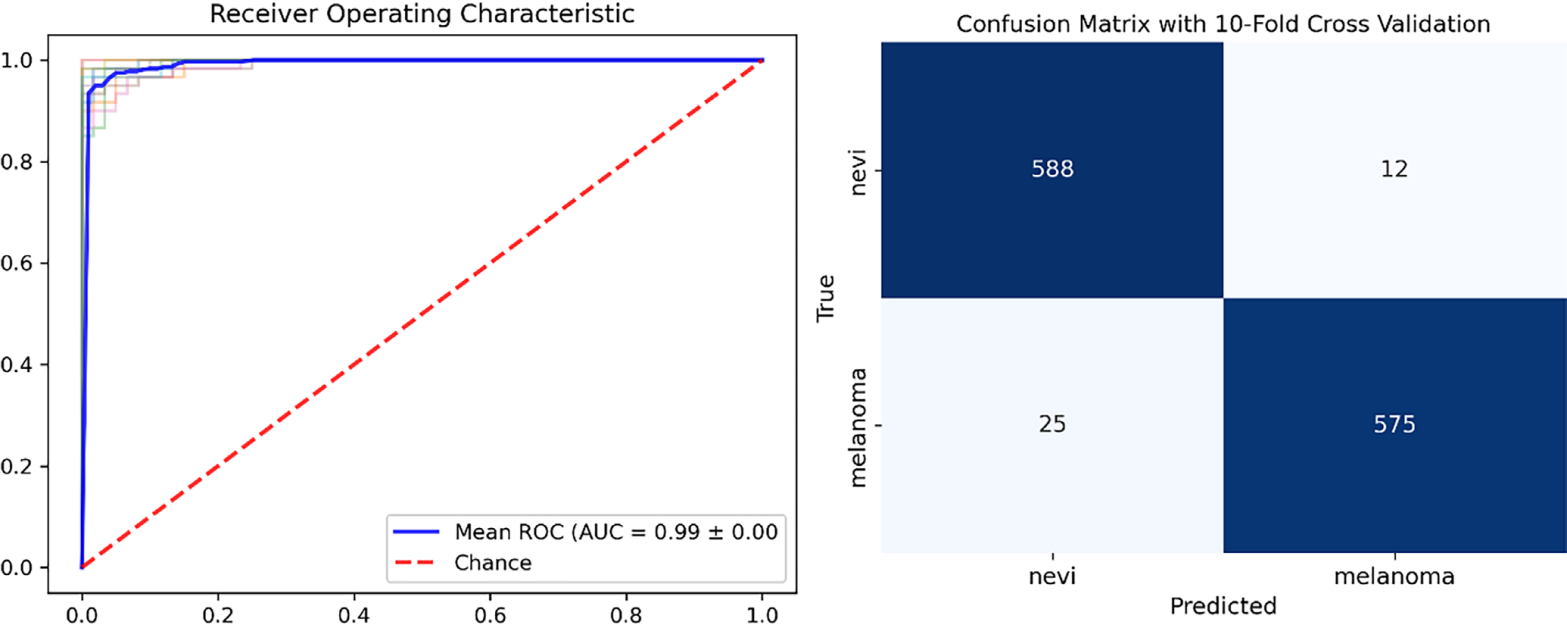
The SVM classification statistics with 10‐fold cross‐validation, with the mean attenuation coefficient, mean *R* ^2^, mean RMSE, and mean maximum A‐scan signal intensity, as well as their standard deviation in each bin as input, yielded an average AUC‐ROC of 0.99 and a classification accuracy of 96.9%.

As stated above, the heatmaps of the attenuation coefficients of nevi and melanoma show clear differences. To further investigate the influence of the attenuation coefficient on distinguishing melanoma from nevi, two sets of SVM classification attempts were made. The first selected only two parameters as inputs, the mean attenuation coefficient and its standard deviation. The second chose all eight parameters as inputs. Figures 5 and 6 show the AUC‐ROCs and confusion matrices after the corresponding 10‐fold cross‐validation, respectively. Specifically, for the group that selected only the mean attenuation coefficient and its standard deviation as input, the overall classification accuracy was 84.3% (1012 of 1200) and the average AUC‐ROC for the 10‐fold cross‐validation was 0.92. For the group that selected all the input parameters, these two figures were 96.9% (1163 of 1200) and 0.99, respectively.

**FIGURE 7:**
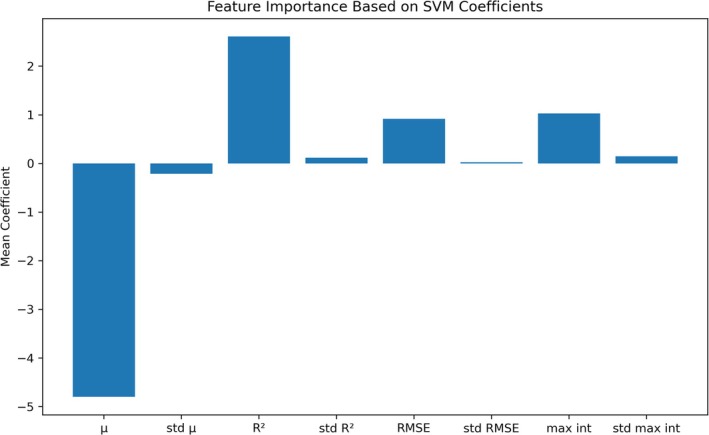
The weight vector derived from the SVM model, incorporating all input variables to assess each variable's contribution to the decision of the model. Variables with larger absolute weight values exert a greater influence on the model's output, where positive or negative coefficients indicate a positive or negative correlation, respectively.

The contribution of each input variable to the decision of the model was quantified by extracting the weight vector from the SVM model, which utilized the attenuation coefficient, *R* ^2^, RMSE, and maximum A‐scan intensity as input variables. The magnitude of each coefficient indicates the impact of the corresponding input variable on the model's decision, with larger absolute values signifying a greater influence. Additionally, the sign of the coefficient (positive or negative) denotes whether the variable is positively or negatively correlated with the model's decision. As illustrated in Figure 7, the primary indicators that affect the classification accuracy of the model remain the attenuation coefficient, *R* ^2^, RMSE, and maximum A‐scan intensity. The standard deviations of these parameters have a lower impact compared to the parameters themselves. For subsequent model optimization and streamlining, it may be beneficial to consider removing these standard deviations as input variables to enhance the model's efficiency without compromising its accuracy.

Notably, the attenuation coefficient emerges as the most significant contributor to the outcome, which also explains why an accuracy of 84.3% can be achieved using only the attenuation coefficient and its standard deviation. The second most influential variable is *R* ^2^, which is intrinsically related to the attenuation coefficient and serves as a critical measure for assessing the appropriateness of the fitted attenuation coefficient. The weights of RMSE and maximum A‐scan intensity are comparable and less impactful than those of the attenuation coefficient and *R* ^2^.

Furthermore, in order to make a correct cancer diagnosis, a meaningful reference indicator is the number of A‐scan instances in each sample that are predicted to be consistent with the sample type, thereby assessing whether more than half of the sample instances are correctly classified as the sample type. As shown in Table 1, when considering the accuracy of the 200 instances in each sample, one can find for the group that only selects the average attenuation coefficient and its standard deviation as input, that the proportion of correctly identified instances for Figure 3 (6) is only 0.45. If this OCT image is used as a basis for clinical diagnosis, the melanoma lesion would be misjudged as non‐melanoma.

**TABLE 1:** The number and proportion of correctly predicted instances for each measurement sample.

Sample info		Group 1 (two inputs)		Group 2 (eight inputs)	
OCT images	Total instance	Correct instance	Correct proportion	Correct instance	Correct proportion
01_Nevus	200	197	0.98	200	1
02_Nevus	200	174	0.87	193	0.96
03_Nevus	200	151	0.76	195	0.97
04_Melanoma	200	200	1	200	1
05_Melanoma	200	200	1	200	1
06_Melanoma	200	90	0.45	175	0.88

When all parameters are selected as input, the correct predicted proportion of the 200 A‐scan instances of Figure 3 (6) reaches 0.88, which means there is at least 88% confidence in correctly assessing the skin lesion as MM through this OCT image. For the other samples, the proportion of instances that were correctly identified is almost 100%. All of these show that using the attenuation coefficient combined with parameters such as *R* ^2^, RMSE, and maximum A‐scan signal intensity, machine learning has considerable potential for correctly identifying melanoma.

### 3.3 Raman Spectra

**FIGURE 8:**
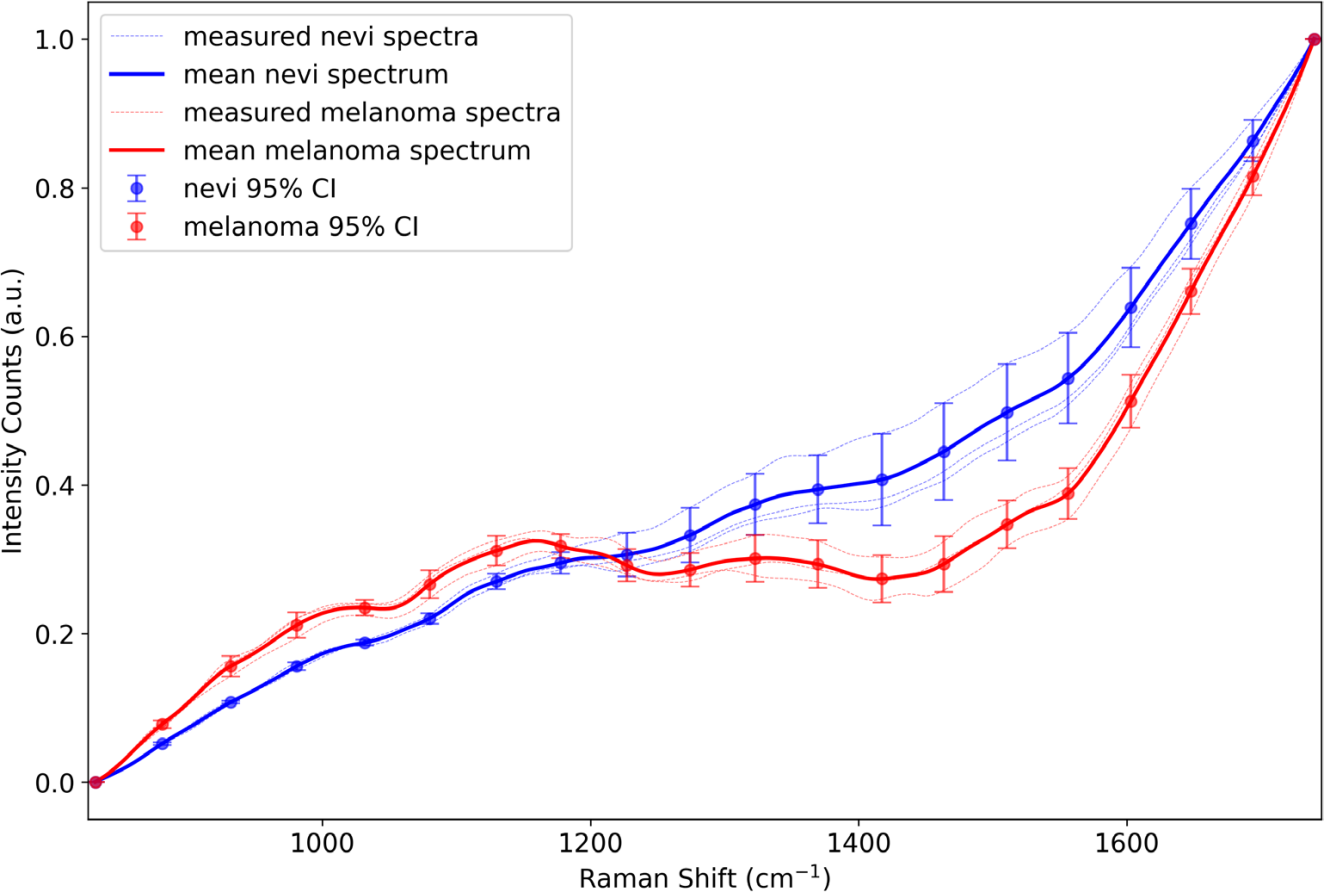
Spectra of benign nevi and malignant melanoma in the fingerprint range of 800–1800 cm^−1^ and their mean spectra, containing autofluorescence backgrounds. 20 points at equal intervals were selected to illustrate their 95% confidence intervals.

Differences in the intensity of the autofluorescence background are notable in the spectral data collected by the spectrometer, as shown in Figure 8. The difference in fluorescence intensity at various Raman shifts may be due to variations in the concentration of common fluorophores with different excitation wavelengths in BN and MMs [20].

**FIGURE 9:**
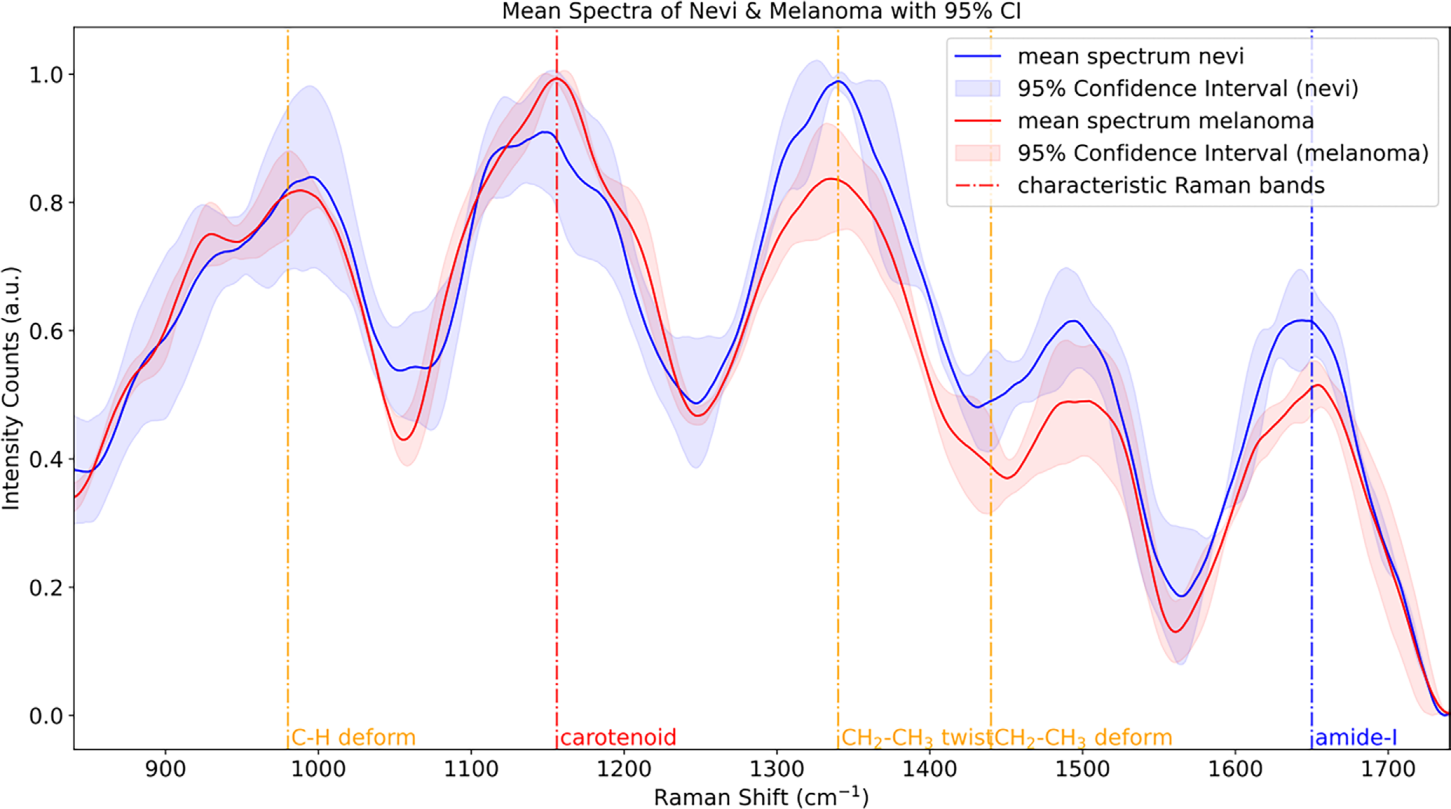
Mean spectra of benign nevi and malignant melanomas after baseline elimination and locations of common Raman bands in the range 800–1800 cm^−1^. 95% confidence intervals are shown over the entire spectral range to explore the intensity differences of specific Raman bands.

**FIGURE 10:**
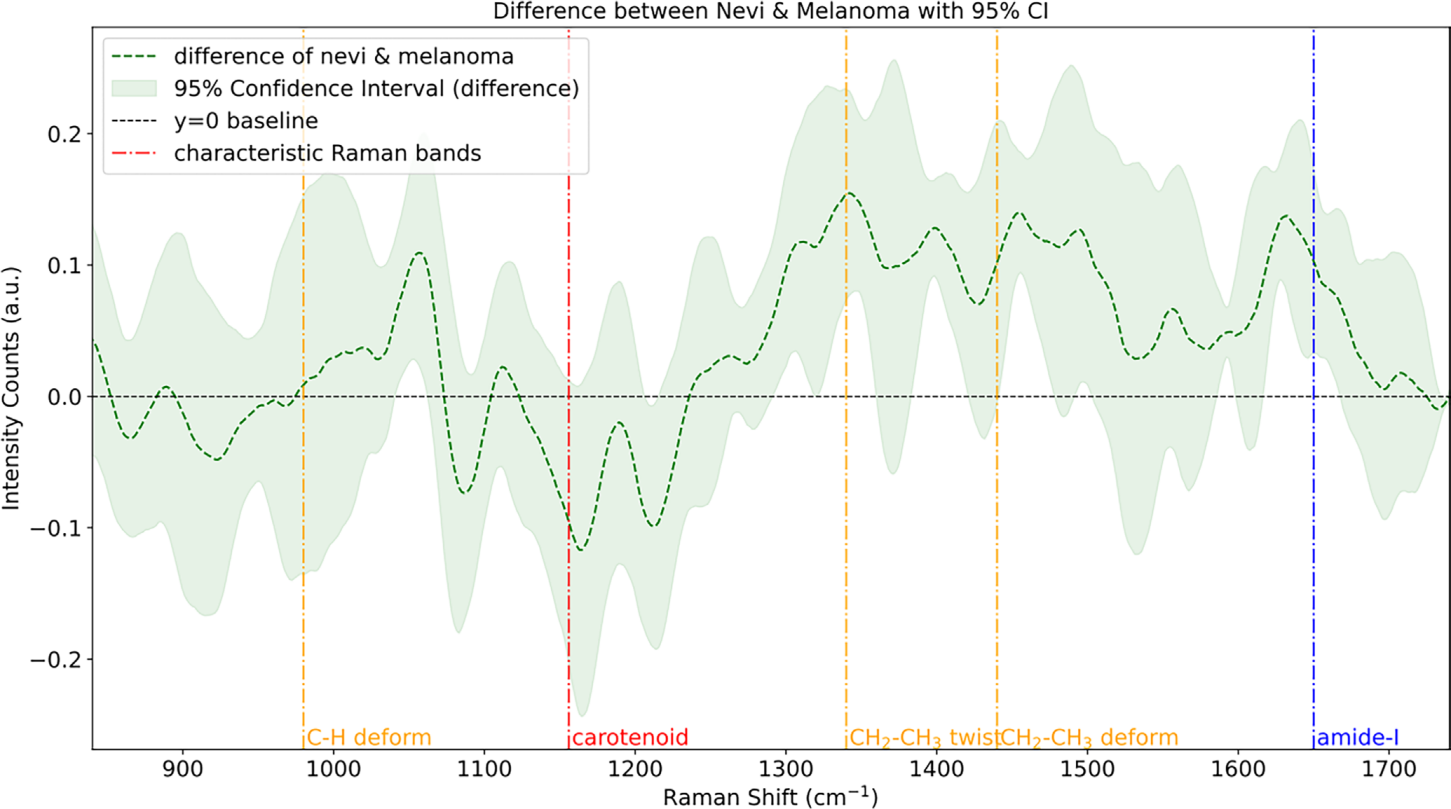
Differences in the mean spectra of benign nevi and malignant melanoma and their 95% confidence intervals, as well as the location of typical Raman bands in the range 800–1800 cm^−1^.

After removing the autofluorescence background, the average Raman spectra of nevi and melanoma, as well as their differences, are shown in Figures 9 and 10, respectively. For nearly identical anatomical locations of the same patient (left and right legs), the intensity of carotenoid lines in melanoma tissue was higher than in BN, reflecting differences in carotenoid concentration between the two types of tissues. Additionally, the Raman spectra of BN showed increased intensities for CH_2_–CH_3_ and amide‐I structures compared to melanoma.

These variations in the intensity of chemical structures are likely indicative of differences in the concentration of common fluorophores associated with skin autofluorescence, such as collagen (peptide bonds in proteins are linked by amide structures) and lipofuscin (comprising lipids and lipoproteins, both of which are rich in CH_2_–CH_3_ structures), and allow for distinctions between melanoma and nevi [21]. Additionally, although carotenoids are not the primary fluorophores contributing to skin autofluorescence, the observed intensity differences in carotenoid content between melanoma and nevi may also influence the variations in their autofluorescence backgrounds at different Raman shifts.

Overall, the observed differences in Raman spectra and autofluorescence background spectra between melanoma and nevi highlight the potential of Raman spectroscopy as a tool for melanoma identification. Furthermore, it demonstrates the capability of providing additional biochemical parameters to support OCT results for a more accurate diagnosis. The next step involves integrating Raman spectral data with various parameters derived from OCT fitting into one machine learning model. This combined approach is expected to further enhance the model's performance and improve the accuracy of cancer diagnosis.

## 4 Conclusions and Outlook

This study explored a novel and fast approach that integrates OCT and Raman spectroscopy to provide both morphological and biochemical insights into skin lesion assessment. The attenuation coefficient derived from OCT A‐scans was obtained via exponential fitting and subsequently employed in a machine learning algorithm to distinguish between BN and MM.

OCT tomographic images exhibited structures similar to histological images, suggesting their potential to deliver complementary information for histological examinations. When only the fitted attenuation coefficient and its standard deviation served as the input variables for SVM classification, an accuracy of 84.3% was achieved. When additional variables, including *R* ^2^, RMSE, and maximum A‐scan intensity, were incorporated, the classification accuracy improved significantly to 96.9%, underscoring the efficacy of the proposed method in melanoma diagnosis.

The Raman spectra along with the autofluorescence background spectra revealed independently notable differences between melanoma and nevi. Combined with the intensity differences of the corresponding Raman bands in the Raman spectra, the variations in autofluorescence intensity were inferred to be attributed to differences in the concentration of fluorophores such as carotenoids, lipofuscin, and collagen in the respective skin tissues.

Future research will aim to expand the sample size of skin lesions and integrate Raman spectroscopy and autofluorescence background data as input variables, alongside OCT data, into the machine learning model. This will enable further validation of the performance of the proposed method and enhance the accuracy of skin cancer diagnosis. Ultimately, the goal is to achieve noninvasive diagnostics of melanoma and other types of skin cancer, minimizing the need for painful biopsies and improving patient care. For this purpose, we are also working on integrating additional modalities such as photoacoustic tomography and high‐frequency ultrasound to provide the relevant information needed for noninvasive and reliable diagnosis of melanoma skin cancer, that is, lesion infiltration depth, morphology, and molecular composition.

## Conflicts of Interest

The authors declare no conflicts of interest.

## Data Availability

The data that support the findings of this study are available from the corresponding author upon reasonable request.
